# Dietary exposure to methyl mercury and PCB and the associations with semen parameters among Swedish fishermen

**DOI:** 10.1186/1476-069X-6-14

**Published:** 2007-05-08

**Authors:** Anna Rignell-Hydbom, Anna Axmon, Thomas Lundh, Bo A Jönsson, Tarmo Tiido, Marcello Spano

**Affiliations:** 1 Division of Occupational and Environmental Medicine and Psychiatric Epidemiology, Department of Laboratory Medicine, Lund University Hospital, SE-221 85 Lund, Sweden; 2 Fertility Centre, Scanian Andrology Centre, Malmö University Hospital, Lund University, SE-205 02 Malmö, Sweden; 3 Section of Toxicology and Biomedical Sciences, ENEA CR Casaccia, Rome, Italy

## Abstract

Dietary POP exposure have shown negative effects on sperm motility and sperm chromatin integrity, as well as an increased proportion of Y-chromosome bearing sperms. However, it has been suggested that in epidemiological studies investigating persistent organochlorine pollutant (POP)-toxicity, other pollutants occurring simultaneously may carry an increased risk of effects, which may obscure a clear interpretation of the role of POP toxicity. One such pollutant is methyl mercury (MeHg), which has been found in fatty fish from the Baltic Sea and as a consequence men with a high consumption of such fish has been found to have twice the MeHg levels compared to men with a low fish consumption.

The aim of the present study was to assess if exposure to MeHg affects male reproductive function, assessed by measuring human sperm motility, sperm concentration, total sperm count, sperm chromatin integrity and the proportion of Y-chromosome bearing sperms. Secondly we also investigated a possible interaction between MeHg and 2, 2', 4, 4', 5, 5'-hexachlorobiphenyl (CB-153), a biomarker for POP exposure, with respect to sperm outcome measures.

Blood and semen samples were collected from 195 Swedish fishermen with a mean age of 47 (range 24–67 years). Blood levels of MeHg ranged from 0.11 to 16.59 μg/L (median 2.25 μg/L) and serum levels of CB-153 from 37 to 1460 ng/g lipid (median 190 ng/g lipid).

The analyses revealed no association between MeHg and any of the outcomes investigated. Although men with low MeHg and high CB-153 had slightly higher DNA Fragmentation Index and fraction of Y-chromosome bearing sperms than men with low levels of both compounds, the effects were not statistically significant. In conclusion, we did not find any associations between MeHg exposure and semen quality or quantity in the dose range observed neither was any synergistic effects between MeHg and CB-153 noted.

## Background

The Baltic Sea, off the east coast of Sweden, has for several decades, been polluted by persistent organochlorine pollutants (POPs), such as polychlorinated biphenyls (PCBs), polychlorinated dibenzo-p-dioxins (PCDDs), polychlorinated dibenzofurans (PCDFs), and dichloro diphenyl trichloroethane (DDT), with its major metabolite dichloro diphenyl dichloroethene (DDE). As a consequence, consumption of fatty fish from the Baltic Sea, such as herring and salmon, constitutes an important exposure source for POPs in the Swedish population [1-3]. Studies on the effects of dietary POP exposure on male reproductive function have shown negative effects on sperm motility [4-7] and sperm chromatin integrity [8]. An increased proportion of Y-chromosome bearing sperm has also been reported [9]. In a majority of these studies 2, 2', 4, 4', 5, 5'-hexachlorobiphenyl (CB-153) has been used as a biomarker for POP, since it correlates very well with total PCB concentration in serum [10] and plasma [11], as well as with the PCB derived dioxin-like effect [11] and the total POP derived dioxin-like effect [12]. Another relevant exposure biomarker is p, p'-DDE, which is present in relatively high serum concentrations in subjects consuming fatty fish from the Baltic Sea [13]. Fatty fish from the Baltic Sea is also a source of exposure to other pollutants, such as methyl mercury (MeHg) [14], and as a consequence men with a high consumption of such fish have been found to have twice the MeHg levels of men with a low consumption [3].

Animal studies suggest an effect on male reproduction of MeHg: from laboratory studies on rats there is evidence that exposure to MeHg impairs spermatogenesis by germ cell deletion via cell-and stage-specific apoptosis [15]. Furthermore, mice exposed to mercury showed a decreased testicular weight [16], and serum testosterone levels were lower than normal in rats treated with MeHg [17]. Monsees et al. [18] studied the *in vitro *effect of mercury on Sertoli cells from rat, and observed that concentrations at non-toxic or slightly cytotoxic concentrations sharply reduced inhibin B production.

Studies in humans have also reported a negative influence of MeHg on human male reproduction. Infertile [19] and subfertile [20,21] men have been found to have higher MeHg levels than fertile men, and Sertoli cell only syndrome and tubular atrophy has been observed among infertile patients with MeHg exposure [22]. Moreover, mercury seminal fluid levels have been found to correlate with abnormal sperm morphology and abnormal sperm motility [23].

Thus, consumption of fatty fish from the Baltic Sea may be a shared exposure route for POPs and MeHg, both found to be hazardous to male reproduction. Indeed, it has been suggested that, in epidemiological studies investigating POP-toxicity, other pollutants occurring simultaneously, such as MeHg, may obscure a clear interpretation of the role of POP toxicity [24]. The difficulty in interpreting results from these studies may occur if the less hazardous compound is acting as a biomarker for the other, but also if there is a synergistic effect of the two chemicals. Results from a recent laboratory experiment suggested that some combinations of concentrations of MeHg and CB-153 have an antagonistic effect on PC12 cells [25]. Moreover, some experimental studies on rats have found synergistic effects on cerebellar functions [26] and brain dopamine [27], although others failed to find impairments on spatial alternation tasks from exposure to both MeHg and PCBs [28]. In humans, studies have suggested that an interaction between PCBs and MeHg may be present with respect to neonatal brain development [29], and cognitive development in preschool children [30]. To the best of our knowledge, no studies have been performed on interacting effects on outcomes related to male reproductive function.

The aim of the present study was to assess if exposure to MeHg affects male reproductive function, assessed by measuring human sperm motility, sperm concentration, total sperm count, sperm chromatin integrity and the proportion of Y-chromosome bearing sperms. Secondly, if there may be an interaction between MeHg and CB-153 with respect to sperm outcome measures.

## Methods

### Study population

Cohorts of fishermen from the Swedish east and west coasts were established in 1988 [3]. In 2000, a postal questionnaire, mainly focused on fracture incidence, was sent to 3,505 west and 1,678 east coast fishermen, born in 1935 or later. The questionnaire included a question about whether the subjects were interested in more information on a study of male semen function. Among the 2,614 subjects (east n = 848 and west n = 1,766) who responded to this specific question, 479 (east n = 171 and west n = 308) wanted more information. These subjects, as well as another 169 east coast fishermen who had become members of the east coast fishermen's organization after the closure of the original cohorts, were contacted and given written detailed information. From the east coast, 130 (38%) men wanted to participate and gave their written informed consent. The corresponding number on the west coast was 136 (44%). During the field study period, another 37 subjects from the west coast and 34 from the east coast were excluded due to logistical reasons, change of mind, sickness or recent vasectomy. Thus, the final number of men included in the present study was 189 (Table 1). Additional details of subject recruitment are given elsewhere [7].

**Table 1:** Distribution of basic semen variables, SCSA results, fraction of Y-chromosome bearing sperms and potential confounders among 189 Swedish fishermen.

	Mean	SD	Median	5–95%
**Exposure variables**				
MeHg (μg/L)	2.9	2.4	2.3	0.5–6.9
CB-153 (ng/g lipid)	240	190	190	62–640
**Outcome variables**				
A+B motile (%)	57	21	62	17–85
Sperm concentration (10^6^/ml)	58	44	48	10–166
Total sperm count (× 10^6^)	184	160	134	23–500
DFI* (%)	19	12	15	6–39
HDS** (%)	10	7	8	4–25
Y-chromosomes (%)	51.2	1.74	51.1	48.7–54.6
**Potential confounders**				
Age (years)	47	9	47	32–60
Abstinence time (days)	3.7	2.6	3.0	1–9
BMI (kg/m^2^)	27	3	27	22–32
Current smokers (%)	23			
Sexually transmitted diseases (%) ***	14			
Disorders in reproductive organs (%) ****	6			
Season of sampling (%)				
Spring	34			
Summer	31			
Autumn	35			
Winter	0			

* DNA Fragmentation Index, ** High DNA Stainable

*** gonorrhoea, Chlamydia, **** varicocele, inguinal hernia, retentio testis

### Non-participants

The non-participants from the original fishermen's cohort had similar age distribution (median 51.8 years, range 29–67) as the participants (median 47.5 years, range 24–67). This was also the case with the fishermen who had become members since 1988 (median 47 years, range 20–67 vs. median 46 years, range 24–59). The fraction of smoking men was 23% among both participants and non-participants.

The men who wanted more information about the semen study had similar body mass index (BMI) values as those who did not want such information (median BMI 27, range 20–37 and 26, 18–44, respectively). However, the median age differed slightly (median 51 years, range 29–67 and 54 years, range 29–67, respectively).

### Questionnaire

Information on lifestyle, and medical and reproductive history was collected through telephone interviews performed by the same person during the whole study. The questionnaire was sent out to the participants a couple of weeks before telephone contact. In this way the subjects had time to get acquainted with the questions. During the telephone contact, an agreement was reached on time and date for collection of semen and blood samples at the subject's home. The participants received information on the procedures for collecting the semen samples both in verbal and written form.

All participants gave their written informed consent and the study was approved by The Ethical Committee at Lund University.

### Mobile laboratory unit

A mobile laboratory unit was established for this study. A mini-van was equipped with all necessary instruments to perform the immediate semen analyses. Phase contrast microscope, 37°C microscope stage heater, centrifuge, incubator, 4-channel cell counter, vortex mixer, Sartorius^® ^scale, PC for CASA (computer assisted sperm analysis) and a freezer/dry ice device was installed.

### Semen sampling and analyses

Semen samples were obtained from 188 men by masturbation into a plastic container. The subjects were requested to have 3–4 days of abstinence before the collection. The median abstinence time was 3.0 days. Only two men had an abstinence time less than one day and five men had an abstinence time of more than 10 days. When a semen sample was collected, the participant completed a form regarding time, date, and the duration of abstinence, spillage, fever and medical treatment during the past 3 months. The sampling was performed between March 2001 and November 2001, and March 2002 and September 2002.

The semen samples were analyzed immediately with respect to semen volume, sperm motility and sperm concentration in the mobile laboratory. All analyses were performed by one person (ARH) and according to guidelines of the World Health Organisation [31]. A detailed description of the procedure has been given elsewhere [7].

The investigator had no knowledge about the exposure biomarker levels of the subjects when she analyzed the semen samples. The Fertility Center is accredited by the European Academy of Andrology and participates in the Nordic Association of Andrology and European Society of Human Reproduction and Embryology (ESHRE) quality control program. The intra-laboratory coefficient of variation (CV) for assessment of sperm concentration was found to be less than 8.5 percent. The average discrepancies for sperm concentration and motility (sum of A + B grade) between the principal investigator (ARH) and the laboratory staff at the Fertility Centre in Malmö were 7 percent and 11 percent, respectively. Previous studies have demonstrated a coefficient of variation among trained technicians within one laboratory, performing analysis of sperm concentration to be 11 percent [32].

#### Sperm Chromatin Structure Assay (SCSA)

The samples were quickly thawed in a 37°C water bath and analysed immediately. The SCSA was applied following the procedure described elsewhere [8,33] In short, cells were stained with acridine orange (AO; Molecular Probes, Eugene, OR, USA), and analyzed by a FACScan (Becton Dickinson, San Jose, California, USA) equipped with an air cooled argon ion laser and standard optical filters to collect green and red fluorescence. A total of 10,000 events were accumulated for each measurement. AO, when intercalated with double-stranded DNA, emits green fluorescence, while AO associated with single-stranded DNA emits red fluorescence. Thus, sperm chromatin damage can be quantified by the flow cytometric measurements of the metachromatic shift from green to red (denatured, single-stranded DNA) fluorescence. In the present study, the extent of DNA denaturation was expressed in terms of DNA Fragmentation Index (DFI), which is the ratio of red to total (red plus green) fluorescence intensity [33].

Additionally, the fraction of high DNA stainable (HDS) cells, which represents immature spermatozoa with incomplete chromatin condensation, was also considered. The percentage of HDS cells was calculated by setting an appropriate gate on the bivariate cytogram and considering those events which exhibit green fluorescence intensity higher than the upper border of the main cluster of the sperm population with a non-detectable % DFI, as immature spermatozoa.

For the flow cytometer set-up and calibrations, a reference semen sample retrieved from the laboratory repository was used. Samples were measured twice during independent flow cytometry sessions and the average value was used. Results from the two measurements were highly correlated (DFI, r = 0.96; HDS, r = 0.96).

#### Y:X sperm chromosome ratio

After thawing and mixing, 10 μl of semen was smeared on to cleaned microscope slides (Superfrost Plus slides, Menzel Gläser, Germany) as described in detail elsewhere [9]. To determine the proportion of X- and Y-chromosomes two-colour fluorescence in situ hybridization (FISH) was performed using PNA (protein-nucleic acid) probes (provided by DakoCytomation, Glostrup, Denmark) targeted against the centromeric region of the X chromosome (Rhodamine-labelled) and the q-arm of the Y chromosome (FITC-labelled).

Slides were examined blindly with an Olympus AX 70 epifluorescence microscope (magnification: 400×) equipped with a single and double band pass filter to detect DAPI, FITC and Rhodamine. The examination was performed blindly, *i. e. *without knowledge of the exposure levels or other subject characteristics. An X or Y chromosome in a sperm nucleus was recognized by a red or a green fluorescent spot, respectively. Sperm nuclei were only scored when morphologically preserved, and sperms showing two signals (disomic or diploidic) were not counted. In every sample the proportion of sperms presenting with a clear red or green signal was ≥ 95%. The median number of sperm nuclei scored per slide in each specimen was 981 (range 276–1917), in 96% being 500 or more. As quality control of the method, in the first part of the study, 500 spermatozoa in 26 slides were assessed by two investigators and in 10 slides twice by the first author. Based on these analyses intra- and inter-observer CVs, as considers the proportion of Y bearing sperms, was estimated to 3.3% and 2.3%, respectively.

### Blood sampling and analysis

Blood samples from 189 men were collected by venepuncture in connection to the collection of the semen samples. The time during the day when the samples were collected varied between 6 AM and 10 PM. Whole blood samples (Venoject, VT-100SH, Leuven, Belgium) were stored at -80° for Hg analysis. Serum from blood samples (BD Vacutainer, SST II Advance; Plymouth, UK) was transferred with ethanol rinsed pasteur pipettes to ethanol rinsed brown glass bottles and special tube and stored at -80°C for POP analysis.

#### Mercury analysis

The determination of total (T)-Hg and inorganic (I)-Hg in whole blood was made in acid-digested samples by cold vapour atomic fluorescence spectrometry [34]. The concentration of MeHg was calculated by subtraction of the concentration of I-Hg from that of T-Hg, assuming that the major part of the organic mercury fraction is in the form MeHg. The limit of detection calculated as three times the standard deviation (SD) for the blanks were for T-Hg and I-Hg in blood, 0.20 and 0.37 μg/L, respectively. The analytical accuracy was checked against reference material. Seronorm trace elements in whole blood (Nycomed Pharma., Oslo, Norway) with recommended values and certified blood samples from Centre de Toxicologie du Quebec, International Comparison Program; Canada (QTC) were used. The obtained result for T-Hg in the Seronorm samples were 1.6 ± 0.19 μg/L (mean ± SD) vs. recommended 1.7 – 2.4 μg/L (N = 20) and for the QTC samples 4.8 ± 0.19 μg/L vs. certified 5.0 μg/L (N = 10). However, there are no reference samples for I-Hg available, but the obtained values for I-Hg in the Seronorm samples (0.72 ± 0.19 μg/L; N = 24) were well in agreement with the results from previous analytical runs of the same batch.

#### CB-153 analysis

The levels of CB-153 were determined as previously described by Richthoff et al. (2003). Briefly, the CB-153 was extracted from the serum by solid phase extraction (Isolute ENV+; IST, Hengoed, UK) using on-column degradation of the lipids and analysis by gas chromatography mass spectrometry. ^13^C_12_-labeled CB-153 was used as an internal standard. The relative standard deviations, calculated from samples analyzed in duplicate at different days, was 7 percent at 0.6 ng/mL (n = 76) and 5 percent at 1.5 ng/mL (n = 37) for CB-153 and the detection limits were 0.05 ng/mL for CB-153. The analyses of CB-153 is part of the Round Robin inter-comparison program (Professor Dr. med. Hans Drexler, Institute and Out-Patient Clinic for Occupational, Social and Environmental Medicine, University of Erlangen-Nuremberg) with analysis results within the tolerance limits.

#### Determination of lipids by enzymatic methods

Plasma concentrations of triglycerides, cholesterol and phospholipids were determined by enzymatic methods using reagents from Boehringer-Mannheim (triglycerides and cholesterol; Mannheim, Germany) and Waco Chemicals (phospholipids; Neuss, Germany). The total lipid concentration in plasma was calculated by summation of the amounts of triglycerides, cholesterol and phospholipids. In these calculations, the average molecular weights of triglycerides and phospholipids were assumed to be 807 and 714. For cholesterol we used an average molecular weight of 571, assuming that the proportion of free and esterified cholesterol in plasma was 1:2.

### Statistical analysis

MeHg was split into quintiles at 1.08, 1.86, 2.79, and 4.40 μg/L (n = 36–37 in each group) and data was analyzed using non-parametric methods. The rationale for this was that the model fit, assessed by checking the distribution of the residuals, was not satisfactory for the original values, for log-transformed values or for the categorized exposure variable. Thus, Kruskal-Wallis' test was used to evaluate differences between exposure groups with respect to the different outcomes. Furthermore, Mann-Whitney's U-test was used to examine differences between the lowest and highest quintile.

CB-153 and MeHg were dichotomized at their respective median (193 ng/g lipid for CB-153 and 2.25 μg/L for MeHg), and the dichotomized variables were then combined so that four groups were established: low MeHg/low CB-153 (n = 56), high MeHg/low CB-153 (n = 34), low MeHg/high CB-153 (n = 35), and high MeHg/high CB-153 (n = 55). A possible interaction between the two exposures was investigated as follows. The relative excess risk due to interaction (RERI), a measure of departure from additivity [35], was calculated for each outcome. Since calculations of RERI depends on risk estimates for each exposure, something that is not available when using non-parametric methods, the outcomes were dichotomized at their respective means in order to produce odds ratios (ORs) for being above the median by using logistic regression. RERI was then estimated using the formula RERI = OR(AB) - OR(AB') - OR(A'B) + 1, where A indicates high CB-153 and A' low CB-153, with the corresponding definition for B and MeHg, and each OR is calculated using the A'B' (i.e. low CB-153 and low MeHg) group as reference. 95% confidence intervals (CIs) for RERI were estimated using the bootstrap percentile 1 method on 500 data sets resampled with replacement from the original data [36].

Correlations were estimated using the Spearman correlation coefficient (r_S_).

Age and abstinence time [33,37-42], BMI [43], season of sampling [44-48], and previous reproductive malformations and diseases [49,50] have all previously been found to be associated with different measures of male reproduction and where thus considered as potential confounders. However, only if a factor was found to correlate (r_S _> 0.20) with both exposure and outcome, the variable was adjusted for. Only age fulfilled this criterion (irrespective of which exposure measure was used).

Analyses were performed using StatXact-6 (Cytel Studio) and SPSS 12.0. The term statistically significant is used to denote a two-sided p-value less than 5%.

## Results

### MeHg and CB-153

**Figure 1:**
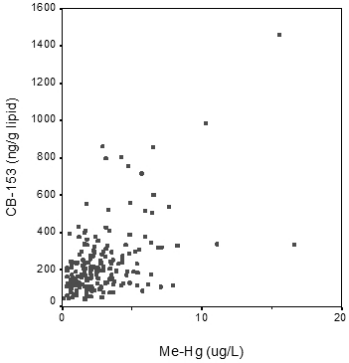
Correlation between serum levels of MeHg and CB-153 among Swedish fishermen (r = 0.51; p < 0.001).

Blood levels of MeHg ranged from 0.11 to 16.59 μg/L (median 2.25 μg/L) and serum levels of CB-153 from 37 to 1460 ng/g lipid (median 190 ng/g lipid). There was a high correlation between MeHg and CB-153 (r_S _= 0.51, p = 0.001, fig. 1).

### MeHg vs. outcomes

**Figure 2:**
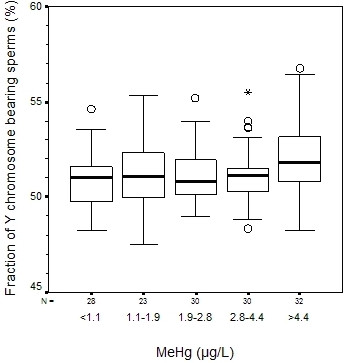
Fraction of Y chromosome bearing sperms (%) for different levels of MeHg. Circles denotes outliers (1.5–3 quartile intervals from the box) and stars denotes extremes (>3 quartile intervals from the box). Please note that the y-axes do not start at zero.

The analyses using Kruskal-Wallis' test revealed no association between MeHg and DFI (p = 0.3), HDS (p = 0.7), fraction of Y-chromosome bearing sperms (p = 0.2), sperm motility (p = 0.9), sperm concentration (p = 0.9), total sperm count (p = 1.0), or semen volume (p = 0.6) (Table 2). The lowest and highest exposure groups differed with respect to the fraction of Y-chromosome bearing sperms (p = 0.03; Figure 2), but after adjustment for age this difference was no longer statistically significant (p = 0.4). No differences between lowest and highest exposure was found for the other outcomes (data not shown).

**Table 2:** Outcome variables (median and 5–95% range) among 189 Swedish fishermen depending on exposure to methyl mercury (MeHg).

			MeHg (μg/L)				
	K-W	J-T	<1.08 (n = 37)	1.08–1.86 (n = 36)	1.86–2.79 (n = 37)	2.79–4.40 (n = 36)	>4.40 (n = 36)

**Exposure**							
MeHg (μg/L)			0.71 (0.28–1.05)	1.59 (1.12–1.85)	2.25 (1.89–2.78)	3.43 (2.85–4.33)	5.92 (4.63–15.54)
CB-153 (ng/g lipid)			125 (48–142)	200 (41–131)	191 (55–353)	230 (76–804)	317 (108–982)
**Outcomes**							
A+B motile (%)	0.90	0.70	62 (17–90)	63 (21–86)	54 (15–83)	64 (14–87)	60 (12–84)
Sperm concentration (10^6/ml)	0.94	0.73	42 (6–119)	54 (4–200)	48 (9–176)	53 (17–207)	48 (16–110)
Total sperm count (× 10^6)	0.98	0.73	135 (14–590)	170 (13–477)	127 (15–732)	159 (26–386)	134 (45–342)
Semen volume (ml)	0.58	0.87	3.5 (0.9–5.9)	3.0 (0.8–5.5)	3.4 (1.2–6.2)	3.1 (0.7–5.8)	3.3 (1.2–7.7)
DFI (%)	0.26	0.05	14 (6–32)	13 (4–36)	17 (6–45)	17 (6–38)	20 (6–44)
HDS (%)	0.71	0.38	10 (3–22)	8 (4–29)	8 (4–30)	7 (4–26)	9 (3–18)
Y-chromosome bearing sperms (%)	0.19	0.04	51.0 (48.7–54.6)	51.1 (47.5–55.2)	50.1 (49.3–54.0)	51.1 (48.8–54.0)	51.8 (48.5–56.4)

Differences between exposure groups are analysed using the Kruskal-Wallis (K-W) test, whereas trends are tried using the Jonckheere-Terpstra (J-T) test.

### Interaction between CB-153 and MeHg

Although men with low MeHg/high CB-153 had slightly higher DFI and fraction of Y-chromosome bearing sperms, none of the age adjusted RERIs were statistically significant (Table 3).

**Table 3:** Outcome variables (median and 5–95% range) among 189 Swedish fishermen, depending on exposure to MeHg and CB-153.

	MeHg/CB-153				
	Low/low (n = 56)	High/low (n = 34)	Low/High (n = 35)	High/high (n = 55)	RERI (95% CI)^a^

**Exposure**					
MeHg (μg/L)	1.07 (0.33–2.10)	3.64 (2.46–7.03)	1.59 (0.35–2.19)	4.01 (2.35–11.10)	
CB-153 (ng/g lipid)	115 (45–180)	126 (76–191)	241 (200–431)	317 (203–861)	
**Outcome**					
A+B motile (%)	65 (24–87)	59 (25–83)	59 (21–80)	57 (12–80)	0.23 (-1.21, 0.94)
Sperm concentration (10^6/ml)	46 (6–195)	48 (10–146)	55 (16–125)	49 (12–192)	-0.99 (-4.28, 0.67)
Total sperm count (× 10^6)	160 (14–591)	125 (26–342)	127 (15–500)	133 (29–473)	0.36 (-1.02, 1.11)
Semen volume (ml)	3.5 (0.9–5.8)	3.3 (0.8–6.2)	2.6 (0.9–7.3)	2.8 (1.1–6.8)	0.40 (-0.58. 0.94)
DFI (%)	13 (4–44)	15 (6–39)	16 (7–39)	20 (7–38)	0.32 (-2.47, 3.54)
HDS (%)	8 (4–24)	9 (4–22)	9 (3–29)	7 (4–26)	-0.74 (-4.02, 0.47)
Y-chromosome bearing sperms (%)	50.9 (48.2–54.6)	51.2 (49.3–54.1)	51.4 (49.9–53.6)	51.4 (48.8–53.9)	-1.54 (-8.99, 2.04)

^a ^Age adjusted RERI (relative excess risk due to interaction) with 95% confidence intervals estimated using the bootstrap percentile 1 method.

Unless otherwise noted, no pairwise comparison reached statistical significance.

## Discussion

The present study gave no support to the hypothesis that exposure to MeHg is hazardous to any of the outcome variables, neither did it provide any support for a synergistic effect between POPs and MeHg.

The low participation rate is a weakness of the study. Although the men had no knowledge of their MeHg and CB-153 levels, they knew that they had been chosen for the study since they were suspected to have a high consumption of fish. Furthermore, even though it is unlikely that the majority of the men have any knowledge of their sperm quality, individuals may know, or suspect, themselves of having low sperm quality if they and their partners have experienced difficulties in conceiving. If, among the 2,614 responders to the original questionnaire, those men with the highest consumption of fatty fish and those with a history of reproductive problems decided not to participate in the study, we run the risk of underestimating a possible association between MeHg and low sperm quality. On the other hand, if men with high fish consumption and reproductive problems are overrepresented in the present study, the risk would be to overestimate a possible association.

Although the studied population was relatively large, the categorization of exposure resulted in groups of only some 30 men. Thus, some of the comparisons may have had insufficient statistical power to detect any differences. However, this categorization method was preferred to be able to detect possible trends with increasing exposure. Moreover, analyses were performed comparing the lowest to highest exposure category, thereby maximising differences in exposure levels.

Due to the relatively strong correlation between CB-153 and MeHg, the joint categorization of the two variables were not optimal, in that the median CB-153 were consistently increasing for the groups low/low – high/low – low/high – high/high, with the corresponding phenomenon for MeHg. Thus, a strong effect of either compound could appear as a synergistic effect between the two. However, since we failed to find such a synergistic effect, the skewed distribution of CB-153 and MeHg in the four exposure groups should not pose a major problem in the present study.

However, the present study has several important strengths. Firstly, the analyst who made the initial semen analyses, sperm motility, sperm concentration and measuring volume was not aware of the participants CB-153 or MeHg levels. Moreover, all analyses were performed by one of the authors (ARH), which is expected to reduce the variation in the assessment of sperm characteristics. As for the other outcome variables, SCSA and FISH were carried out in complete blindness towards exposure. Finally, all the interviews were made by one person.

In the present study, the correlation between MeHg and CB-153 was reasonably high (r_S _= 0.51). This is in agreement with previous studies were the coefficient of correlation between mercury and CB-153 in blood varied between 0.35 and 0.47 [24,51]. When interpreting these correlation coefficients, one must bear in mind that the two compounds behave differently in the human body: Whereas MeHg in general is present as water-soluble complexes and does not distribute to fatty or lipid regions in the body but accumulates to a higher degree in the muscles, lipid rich tissue is the most important storage site for POPs [52].

The blood levels of MeHg in the present study ranged from 0.1 to 17 μg/L, with a median at 2.25 μg/L, and represented on average 86% of total Hg level (data not shown). Thus, the levels are in the same order of magnitude as those found by Chia et al [53], who failed to find any difference in blood mercury between men with <40% motile sperm compared to those with ≥ 40% (1.8 μg/L vs 1.91 μg/L). In studies of men with higher levels of blood mercury, infertile men were found to have higher levels than the control group (8.1 μg/L vs 6.2 μ/L [assuming that the unit "mmol/L" was a misprint in the paper and should really be "nmol/L"]) [19], and men with blood mercury higher than 10 μg/L had reduced sperm concentration, percentage of morphologically normal sperm, and percentage of motile sperm, although the differences were not statistically significant [54] Furthermore, in a population of men with mean blood mercury concentration of 8.3 μg/L, seminal fluid concentrations of mercury were found to correlate with abnormal sperm morphology and abnormal sperm motion, although not with the overall percentage of motile sperm or sperm concentration [23]. Other studies have measured mercury in urine or hair rather than blood. The levels of mercury are therefore not easily compared to those in the present study. However, the results from the studies on hair mercury suggest subfertile men to have higher concentrations of mercury than fertile men [20,21], whereas a study on mercury in urine failed to find a correlation between mercury concentrations and semen quality [55].

In some studies, interacting effects of MeHg and PCBs have been found with respect to neurobehavioral endpoints in children [29,30]. However, the literature on this topic is scarce, and we have not been able to find any studies investigating an interacting effect with respect to male reproductive parameters.

In a previous study concerning POPs and male reproduction, a threshold-like effect was noted when categorizing CB-153 serum levels into equally sized quintiles [8]. A significantly lower DFI was found in the lowest CB-153 quintile compared to the other quintiles. The lowest quintile was then defined as having less than 113 ng/g lipid, which is lower than the cut-off used to define low and high CB-153 in the present study (193 ng/g lipid). Using this cut-off in the present analysis, the results were however similar to those presented (data not shown). Thus, the differences in the findings should not be the result of the low exposure group being diluted in the present study.

## Conclusion

In conclusion, we did not find any associations between MeHg exposure and semen quality or quantity, at least in the dose range observed, neither was any synergistic effects between MeHg and CB-153 noted.

## Competing interests

The author(s) declare that they have no competing interests.

## Authors' contributions

A R-H collected all samples and carried out some of the semen analysis. Drafted the manuscript and performed some of the statistical analysis. A.A performed the statistical analysis and helped to draft the manuscript. T.L carried out the mercury analysis. B.AG. J was responsible for the organochlorine analysis. T.T performed the FISH analysis. M.S performed the SCSA analysis and helped to draft the manuscript. All authors have read and commented on the manuscript.
